# The importance of early recognition of extraintestinal manifestations of digestive tract dysfunction following gastrointestinal surgery

**DOI:** 10.1093/jscr/rjae326

**Published:** 2024-05-24

**Authors:** Michelle Pang, Scott Kuwada

**Affiliations:** John A. Burns School of Medicine, University of Hawaii at Manoa, Department of Medicine, Honolulu, HI 96813, United States; John A. Burns School of Medicine, University of Hawaii at Manoa, Department of Medicine, Honolulu, HI 96813, United States; The Queen’s Medical Center GI Services and Liver Center, Department of Gastroenterology, Honolulu, HI 96813, United States

**Keywords:** gastrojejunostomy, peripheral neuropathy, dry beri beri

## Abstract

We report a case of a 47-year-old male who presented with altered mental status. A review of his records revealed a weight loss of 20 lbs over the past 6 years, a recent hospitalization for idiopathic polyneuropathy with failure to thrive, and prior surgeries for peptic ulcer disease and small bowel obstruction. He was alert but had retrograde amnesia and peripheral neuropathy. A diagnosis was made, and the patient improved with treatment but was unfortunately left with irreversible neurological deficits. We discuss the importance of recognizing the extraintestinal manifestations of gastrointestinal dysfunction following gastrointestinal surgery.

## Introduction

Gastrojejunostomies involve the creation of an anastomosis between the stomach and the jejunum, bypassing portions of the small bowel rich in metabolizing enzymes. This may compromise the absorption of both macro- and micronutrients [1]. Prompt recognition of clinical syndromes associated with resulting nutritional deficiencies is crucial to the prevention of long-term consequences. We present a case of nutritional deficiency following gastrojejunostomy, leading to significant neurological sequelae.

## Case report

A 47-year-old male, with a history of protein C deficiency on Warfarin and a seizure disorder, was brought to the Emergency Department (ED) with altered mental status after being found on his bedroom floor. The patient’s mother reported that he had been chronically ill for several years. He was walker-dependent but cognitively intact prior to admission, with no history of falls. He was, however, recently hospitalized for idiopathic polyneuropathy with failure to thrive. On the exam, the patient was somnolent and oriented only to people. His neurologic status declined, and he developed hypoxemic respiratory failure, subsequently undergoing intubation.

A head computed tomography (CT) in the ED revealed a 1.6 cm left acute on chronic subdural hematoma with mass effect and a 1.9 cm left to right midline shift. There were scattered areas of subarachnoid hemorrhage and a right frontal lobe encephalomalacia unchanged from the previous brain MRI. A spot EEG showed no seizure activity. Labs showed an INR of 4.1 and an Hgb of 8.8 g/dl. He received prothrombin complex concentrate and Vitamin K for Warfarin reversal, and the subsequent INR was 1.2. He underwent burr hole evacuation with significant improvement of the hematoma and midline shift.

CT abdomen and pelvis showed probable postsurgical changes suggesting gastrojejunostomy, as well as postsurgical changes within the cecum and bowel loops in the left abdomen. The liver had a lobulated contour, but there were no liver masses, splenomegaly, varicosities, or ascites. A chest CT demonstrated no lung masses but showed patchy ground glass opacities in the right upper and lower lobes with a small right pleural effusion.

On hospital Day 5, gastroenterology was consulted due to evidence of severe malnutrition. The patient denied abdominal pain, chronic diarrhea, nausea, or vomiting. The patient’s mother reported that he had a surgery for peptic ulcer disease over 20 years prior to admission, as well as a surgery for bowel obstruction 5 years prior to admission. A physical exam revealed a midline abdominal scar and midline hernia, but no hepatosplenomegaly or jaundice. He was alert and oriented to person, place, and time, but had retrograde amnesia and stocking and glove neuropathy.

The following day, he underwent esophagogastroduodenoscopy, which confirmed a gastrojejunostomy with patchy erythematous mucosa in the gastric body and antrum and edematous mucosa in the duodenum. The examined jejunum was normal. Biopsies of the gastric body, antrum, and duodenum were normal. Whole-body Vitamin B1 was found to be 18 nmol/L (normal 78–185). He was treated with intravenous thiamine and later transitioned to oral thiamine. His neurological symptoms improved upon discharge, though he still had decreased lower extremity sensation and ataxia. He was discharged to an acute rehabilitation center in stable condition.

## Discussion

We report a case of non-alcoholic dry beriberi following gastrojejunostomy for peptic ulcer disease. Thiamine (Vitamin B1) is essential for carbohydrate and amino acid metabolism as well as nerve impulse propagation [1]. Thiamine has limited storage and a short half-life of 9–18 days, so consistent intake is crucial to maintain levels necessary for proper metabolic functioning [1, 2]. Severe deficiency can lead to ‘dry’ or ‘wet’ beriberi and Wernicke–Korsakoff syndrome in severe cases. Thiamine deficiency is rare in the United States and typically occurs with chronic alcohol use, but is also associated with malnutrition, malabsorption, and increased utilization [2]. Thiamine deficiency has also been recognized as a complication following gastrointestinal (GI) surgeries for morbid obesity [3, 4] and cancer [5].

Various clinical syndromes occur in the setting of thiamine deficiency, with dry beriberi encompassing the potential neurologic manifestations. This includes sensory and motor peripheral neuropathy, muscle atrophy, and paralysis. Severe cases can lead to Wernicke–Korsakoff syndrome, with Wernicke encephalopathy presenting as a triad of confusion, oculomotor dysfunction, and gait ataxia, and Korsakoff syndrome as confabulations, anterograde and retrograde amnesia, personality changes, and psychosis [6]. Thiamine supplementation typically causes rapid improvement, but untreated thiamine deficiency can cause permanent neurologic impairment or be fatal [2, 7].

Thiamine is absorbed in the proximal small intestine through both active transport and passive diffusion via transporters ThTr1 and ThTr2 [8]. The majority of thiamine in serum is protein-bound to albumin, with over 90% contained within erythrocytes. Thiamine deficiency is a well-documented complication following GI surgery [4, 9–12] and thus should be suspected in postsurgical patients developing peripheral neuropathy. In our case, the patient’s gastrojejunostomy bypassed a portion of the proximal small intestine, leading to a loss of effective absorption of thiamine. Unfortunately, he did not seek medical attention when his paresthesias began.

Gastrojejunostomies are performed for different indications, including tumors, trauma, or complicated peptic ulcer disease, as in this case. Thiamine deficiency is a known complication following gastrojejunostomy, likely due to malabsorption [9]. Other important cofactors and vitamins, such as iron, Vitamin D, calcium, and pyridoxine, are absorbed principally in the proximal small intestine and may be deficient in patients following gastrojejunostomy [13]. In fact, our patient was later found to be deficient in iron and demonstrated osteoporosis.

Although gastrojejunostomy may lead to increased thiamine demand, which can exacerbate underlying thiamine deficiency [11], our patient’s neurologic symptoms developed several years after surgery. Thus, his thiamine deficiency was most likely related to both alterations in absorption and poor oral intake. He had significant improvement in his symptoms after treatment but has permanent neurologic sequelae.

In patients with neurologic abnormalities and a history of surgical alterations in upper GI anatomy, thiamine deficiency should be considered as a cause. Given the risks associated with long-term untreated thiamine deficiency, further investigation into early supplementation in symptomatic patients with risk factors may be beneficial [14]. Prompt recognition and treatment of thiamine deficiency is critical for the prevention of permanent sequelae in patients who have undergone upper GI tract surgery, leading to diversion of nutrients away from the proximal small intestine.
